
JOURNAL INFORMATION
==============================
NLM Title Abbreviation: Atlanta J Rec Med
NLM Journal ID: 0001011

Atlanta Journal-Record of Medicine

Publisher: Biomedical Journal Digitization Project

ARTICLE INFORMATION
==============================
PMCID: PMC9070223
PMID: 36020878
Article version: 1
Subjects: Medical Items

Medical Items

Print publication date: 1910 Jun
Volume: 56
Issue: 3
First page: 166
Last page: 166
License: This work is free of known copyright restrictions. For more information, please see PMC Back Issue Digitization.
License URL: https://creativecommons.org/publicdomain/mark/1.0/

==============================

### MEDICAL ITEMS.

### ETHYL CHLORIDE (KELENE).

Has now been in constantly increasing use in general hospitals for nearly four years, in general anæsthesia, largely as adjuvant to ether and chloroform, the mehtod of administration being by inhalation, the drug being sprayed on a mask covered by a number of layers of gauze, until the patient loses consciousness.

"The exponents of this method claim that greater safety is obtained, as the various stages of anæsthesia can be better followed, than when a rapid induction compresses the different stages into fifty or sixty seconds, making it almost impossible to differentiate them.

"As an adjuvant before the use of ether or chloroform, it fills a gap in the armamentarium of the anæsthetist between the light and brief anæsthesia of nitrous oxide and the deep narcosis of chloroform and ether."

For spraying, no apparatus equals the Glass Automatic Spraying Tube, of Fries Bros., 92 Reade Street, New York, with "Kelene" (pure chloride of ethyl).

---

### THE SECOND SUMMER.

Experience has shown that during the second or "teething summer" weakened stomachs are strengthened, faulty metabolism is corrected, fatigued heart and circulation is supported, and many a tired, wornout nervous system is restored to its proper tone by the systemic and intelligent use of small doses, 20-30 drops, according to age, of Gray's Glycerine Tonic Comp.

............TREATMENT OF...........
**MORPHINE, OPIUM,**
**WHISKEY, COCAINE**
AND ALL DRUG AND NERVOUS DISEASES.

**DR. W. A. STARNES**
**SANITARIUM.**
Telephone Decatur 94.
P. O. Box 269 Atlanta, Ga.
